# Genomic Predictions With Nonadditive Effects Improved Estimates of Additive Effects and Predictions of Total Genetic Values in *Pinus sylvestris*

**DOI:** 10.3389/fpls.2021.666820

**Published:** 2021-07-07

**Authors:** Ainhoa Calleja-Rodriguez, ZhiQiang Chen, Mari Suontama, Jin Pan, Harry X. Wu

**Affiliations:** ^1^Skogforsk (The Forestry Research Institute of Sweden), Sävar, Sweden; ^2^Department of Forest Genetics and Plant Physiology, Umeå Plant Science Centre, Swedish University of Agricultural Science, Umeå, Sweden; ^3^Beijing Advanced Innovation Centre for Tree Breeding by Molecular Design, Beijing Forestry University, Beijing, China; ^4^National Research Collection Australia, CSIRO, Canberra, ACT, Australia

**Keywords:** scots pine (Pinus sylvestris L), genomic prediction, nonadditive effects, dominance, epistasis, response to selection, genetic gain

## Abstract

Genomic selection study (GS) focusing on nonadditive genetic effects of dominance and the first order of epistatic effects, in a full-sib family population of 695 Scots pine (*Pinus sylvestris* L.) trees, was undertaken for growth and wood quality traits, using 6,344 single nucleotide polymorphism markers (SNPs) generated by genotyping-by-sequencing (GBS). Genomic marker-based relationship matrices offer more effective modeling of nonadditive genetic effects than pedigree-based models, thus increasing the knowledge on the relevance of dominance and epistatic variation in forest tree breeding. Genomic marker-based models were compared with pedigree-based models showing a considerable dominance and epistatic variation for growth traits. Nonadditive genetic variation of epistatic nature (additive × additive) was detected for growth traits, wood density (DEN), and modulus of elasticity (MOEd) representing between 2.27 and 34.5% of the total phenotypic variance. Including dominance variance in pedigree-based Best Linear Unbiased Prediction (PBLUP) and epistatic variance in genomic-based Best Linear Unbiased Prediction (GBLUP) resulted in decreased narrow-sense heritability and increased broad-sense heritability for growth traits, DEN and MOEd. Higher genetic gains were reached with early GS based on total genetic values, than with conventional pedigree selection for a selection intensity of 1%. This study indicates that nonadditive genetic variance may have a significant role in the variation of selection traits of Scots pine, thus clonal deployment could be an attractive alternative for the species. Additionally, confidence in the role of nonadditive genetic effects in this breeding program should be pursued in the future, using GS.

## 1. Introduction

Additive genetic variance is the main source of the variation contributing to selection response in breeding programs (Hem et al., 2021). Narrow-sense heritability, as a measurement of the proportion of additive genetic variation over the phenotypic variation, contributes to selection efficiency in tree breeding programs in terms of accuracy of breeding values (White et al., 2007). Many advanced forest tree breeding programs use clonal and/or full-sib family tests in their selection strategies, where the resemblance between related individuals consists not only of additive genetic variation but also nonadditive genetic variation (i.e., dominance and epistatic effects) (Falconer and Mackay, 1996; Lynch and Walsh, 1998). Indeed, the Swedish *Pinus sylvestris* L. (Scots pine) breeding strategy uses full-sib mating with forward selection and progeny testing, where clonal testing is of great interest because it enables more efficient selection using clonal cuttings as propagation method (Andersson and Lindgren, 2011; Rosvall, 2011).

Ignoring nonadditive genetic effects would lead to inaccurate estimates of narrow-sense heritability and consequently less precise estimates of breeding values and reduced genetic gains (Lebedev et al., 2020). For instance, if the total variation is expected to be defined more precisely into its additive, nonadditive, and residual variances, thus more specific discerning would lead to more accurate estimations of heritability and breeding values (Walsh and Lynch, 2018). The role of nonadditive genetic effects has been widely studied in forest trees with pedigree-based methods (Foster and Shaw, 1988; Mullin and Park, 1992; Isik et al., 2004; Baltunis and Brawner, 2010; Chen et al., 2020) and has been recommended to use for clonal forestry (Rosvall et al., 2019). In a *Pinus radiata* L. (radiata pine) study, the diameter at breast height at age five showed a large amount of epistatic variance (Baltunis et al., 2009). However, epistatic effects did not have a significant effect on growth in *Pinus taeda* L. (loblolly pine) (Baltunis et al., 2007), but contributed to fusiform rust (Isik et al., 2003), whereas dominance variance was considerable in the volume variation at age six (Isik et al., 2003) and somewhat large on early growth in clonal and seedling full-sib populations (Baltunis et al., 2007).

Low estimates of nonadditive genetic variation may not necessarily mean this source of variation is not present since a suitable family structure and a proper mating design are also needed to obtain reliable estimates of nonadditive genetic effects (Munoz et al., 2014; Chen et al., 2020). Also, the lack of a good mating design would lead to a poor estimation power of variance components (Framton and Huber, 1995; Pégard et al., 2020) and attention should be paid when exploiting full-sib family structures. The role of dominance effects in many clonal and full-sib breeding programs is well studied, whereas less is known of epistatic variance. It has been, however, argued that epistatic variation generally has only a little contribution to the quantitative trait variation relative to additive genetic variance (Hill et al., 2008) and is only a concern when dealing with individual genes or gene pairs (Mäki-Tanila and Hill, 2014).

The lack of consideration of nonadditive genetic variation in genetic evaluation can be also explained by several challenges in the estimation of variance components regarding family structure and mating design (Hem et al., 2021). The defiance of using a full-sib family structure in genetic evaluation is that nongenetic effects are often confounded with genetic effects (Baltunis et al., 2007; Lee et al., 2010) and simultaneously dominance and epistatic effects are confounded with additive genetic and nongenetic effects (Lee et al., 2010; Bouvet et al., 2016). A large proportion of the variance due to allele interactions can be considered as additive genetic variance if the additive and nonadditive genetic effects are not orthogonal, and an indication of confounding can be seen when variance components are being highly correlated (Lynch and Walsh, 1998; Hill et al., 2008; Hill, 2010).

Genomic marker-based models offer more in-depth possibilities to investigate the subjacent variation of complex traits, by allowing to account for the effect of nonadditive genetic components (Su et al., 2012; Munoz et al., 2014; Varona et al., 2018). Many studies using genomic marker-based information have been already performed in several forest species, though only a few of them have evaluated the partition of genetic variance into additive and nonadditive components (Grattapaglia et al., 2018; Lebedev et al., 2020). However, in animal and plant breeding, it has been more widely studied (Vitezica et al., 2013, 2017, 2018; Aliloo et al., 2016; Piaskowski et al., 2018; Ferrão et al., 2020).

More accurate genetic parameters were estimated by using genomic marker-based information with additive and nonadditive relationship matrices to evaluate genetic covariances between relatives in *Picea abies* L. Karst (Norway spruce) (Chen et al., 2019), *Picea glauca* (Moench) Voss (white spruce) (El-Dien et al., 2016), or *Eucalyptus urophylla Eucalyptus grandis* (eucalypt hybrids) (Bouvet et al., 2016; Tan et al., 2018). The use of genome-wide marker information will enable to distinguish the additive from the nonadditive components when using different family structures like open-pollinated and non-clonal populations, which is not possible with pedigree-based methods and without a specific mating design (El-Dien et al., 2018). In a loblolly pine population a considerable improvement in breeding value estimation for height was notice when using genomic marker-based relationship matrix, as a result of genetic variances partitioning more precisely into additive, dominance and first order epistatic effects (Munoz et al., 2014). In particular for this loblolly pine population and trait, additive and nonadditive genetic variances were of similar magnitude (Munoz et al., 2014).

Genomic selection (GS) is expected to accelerate the rate of genetic improvement of Norway spruce and Scots pine in Sweden, one of the largest forest producer of the world (Chen et al., 2018; Calleja-Rodríguez et al., 2020; Zhou et al., 2020). Accuracy of GS and genomic predictions depends, among others, on the effective population size but also on the size of the population used to train the genomic model, the level of linkage-disequilibrium (LD), the heritability of the phenotypic trait evaluated, density and amount of the single nucleotide polymorphism (SNP) markers, distribution of the quantitative trait loci (QTL) effects, and the genomic model used (Goddard, 2009; Lenz et al., 2017; Li and Dungey, 2018). To date, for Scots pine, the first genomic prediction study comparing pedigree-based (pedigree best linear unbiased prediction or PBLUP), with a genomic best linear unbiased prediction (GBLUP), Bayesian-LASSO (BL), and Bayesian Ridge Regression (BRR), using genotyping-by-sequencing (GBS) data, showed an improved efficiency to estimate additive genetic values of all genomic-based models (Calleja-Rodríguez et al., 2020).

Well studied benefits of GS for forest tree breeding programs, in increasing genetic gains by shortening generation intervals and increasing prediction accuracies (Grattapaglia and Resende, 2011; Iwata et al., 2011; Grattapaglia et al., 2018), and combining the methodology with clonal propagation and somatic embryo-genesis (Li and Dungey, 2018), can be utilized in the Swedish conifer breeding programs to decrease generation intervals. Another advantage of GS in forest tree breeding is the possibility to increase the selection intensities and getting larger genetic gains, by reducing the breeding cycle through skipping or reducing the field-testing periods (Harfouche et al., 2012; Isik, 2014; Grattapaglia et al., 2018). More precise estimates of breeding values are possible with GS by using genome-wide markers to generate the genomic realized relationship matrix (VanRaden, 2008), which estimates kinships between individuals more accurate based on the actual fraction of the genome shared that is identical by descent or by state (White and Hill, 2020). Furthermore, genome-wide markers also can consider the within family variation in the Mendelian sampling term, specifying relationships among individuals that include simultaneously contemporary and historical pedigrees (Ødegård and Meuwissen, 2014, 2015; Grattapaglia et al., 2018). Without a doubt, GS combined with flowering stimulation will accelerate the rate of genetic improvement in Scots pine where the generation interval ranges from 20 to 30 years, including a field-testing phase of 12–15 years (Calleja-Rodríguez, 2019; Calleja-Rodríguez et al., 2020).

The current study was initiated to have the first indication of nonadditive genetic effects using genomic marker data on the Scots pine breeding population and can be used as a basis in building GS models for the breeding program. The objectives were to implement nonadditive genetic effects including dominance and epistatic effects in genomic prediction models in Scots pine, for growth, wood stiffness, and wood density (DEN). In addition, the response to the selection of three GBLUP and two PBLUP models used was also evaluated, and genetic gains of early GS and conventional pedigree selection compared.

## 2. Materials and Methods

### 2.1. Plant Material and Phenotypes

In this study, a Scots pine full-sib progeny trial was used (named as F261-Grundjärn). The trial was established in 1971 for the Swedish tree improvement program at Skogforsk (the Forestry Research Institute of Sweden) and was described in Fries (2012). In brief, 695 progeny trees (F1-generation) from 184 full-sib families, generated from a partial diallel mating design of 40 plus trees (F0-generation), were assessed for growth and wood properties. The traits measured were tree height at ages 10 and 30 (Ht1 and Ht2, respectively), diameter at breast height at ages 30 and 36 (DBH1 and DBH2, respectively), microfibril angle (MFA), static modulus of elasticity (MOEs) and wood mean density (DEN), obtained from Silviscan (RISE AB, Stockholm, Sweden) analyses, and dynamic modulus of elasticity (MOEd) predicted by Hitman ST300. Growth and wood traits were described in detail in Calleja-Rodríguez et al. (2020) and Hong et al. (2014), respectively.

### 2.2. Genotypes

An exhaustive description of the GBS library preparation, SNP markers filtering, and calling was already described in Calleja-Rodríguez et al. (2020). In summary, genomic DNA from vegetative buds or needles from the progeny and parent trees (827 samples with replicates included) were used to prepare three GBS libraries that were sequenced on an Illumina HiSeq 2000 platform at SciLifeLab (Sweden). After filtering and calling, 24,152 informative SNP markers were kept.

#### 2.2.1. Single Nucleotide Polymorphism Imputation

A baseline imputation of missing genotypes was first performed through the LD K-nearest neighbor method (Money et al., 2015) in TASSEL (Bradbury et al., 2007). Additionally an extra random imputation was done with the function codeGeno from the synbreed package (Wimmer et al., 2012) in R (R Core Team, 2019), which filtered out SNPs with minor allele frequency (MAF) lower than 1%, resulting in the retention of 6344 SNPs.

### 2.3. Statistical Analysis

#### 2.3.1. Initial Analysis

Adjusted phenotypes were obtained by removing either the spatial auto-correlation or variation due to design effects from the data, to obtain within-trial environmentally adjusted phenotypic data as described by Calleja-Rodríguez et al. (2020). The adjusted phenotypic data were used in the current study.

#### 2.3.2. Genomic- and Pedigree-Based Best Linear Unbiased Predictions

The complete data set was used to estimate variance components for each trait using the PBLUP and GBLUP methods. Two univariate linear mixed models which included either additive (PBLUP-A and GBLUP-A) or additive and dominance (PBLUP-AD and GBLUP-AD) genetic effects were used. In addition, for GBLUP, a third model including additive, dominance, and epistatic genetic effects (GBLUP-ADE) was used.

#### 2.3.3. Additive, Dominance, and Epistatic Relationship Matrices

The pedigree-based additive numerator relationship matrix (**A**) and the dominance numerator genetic relationship matrix (**D**) were estimated according to Lynch and Walsh (1998) based on pedigree information. Briefly, the diagonal elements (i) of **A** were calculated as $A_{ii}=1+f_{i}=1+\frac{A_{gh}}{2}$, where *g* and *h* are the parents of individual *i*'s. The relationship between individuals *i*th and *j*th are the off-diagonal elements and were estimated for **A** as $A_{ij}=A_{ji}=\frac{A_{jg}+A_{jh}}{2}$, and for **D** as $D_{ij}=D_{ji}=\frac{A_{gk}A_{hl}+A_{gl}A_{hk}}{4}$, where *k* and *l* are the *j*'s parents.The diagonal elements of **D** are *D*_*ii*_ = 1. ASReml v4.1 (Gilmour et al., 2015) and ASReml-R (Butler et al., 2009) were used to estimate **A**, while function kin from the synbreed (Wimmer et al., 2012) package within the R statistical environment (R Core Team, 2019) was used to estimate **D**.

The genomic-based additive relationship matrix (**G_*A*_**), called realized relationship matrix was estimated as, $G_{A}=\frac{ZZ^{\prime }}{2\sum_{i=1}^{m}p_{i}(1-p_{i})}$ (VanRaden, 2008), where **Z** is rescaled genotype matrix following **M−P**; where **M** is the genotype matrix with genotypes coded as 0, 1, and 2 according to the number of alternative alleles and with dimensions number of individuals (*n*) by number of loci (*m*); **P** is the matrix of locus scores 2*p*_*i*_, with *p*_*i*_ being the *i*th allele frequency and $2\overset{p}{\underset{i=1}{\sum }}p_{i}\left(1-p_{i}\right)$ is the variance of markers summed across loci. The genomic-based dominance relationship matrix (**G_*D*_**) was estimated following Vitezica et al. (2013) as $G_{D}=\frac{WW^{\prime }}{\sum_{i=1}^{m}4p_{i}^{2}q_{i}^{2}}$ where **W** is the matrix containing $-2q_{i}^{2}$ for the alternative homozygote, 2*p*_*i*_*q*_*i*_ for the heterozygote, and $-2p_{i}^{2}$ for the reference allele homozygote of the *i*th SNP.

The genomic-based relationship matrices based on first-order epistatic interaction were calculated by the Hadamard product, cell by cell multiplication represented by ⊙ and trace (*tr*) (Vitezica et al., 2017, 2018). The additive × additive terms were estimated $G_{AA}=\frac{G_{A}⊙G_{A}}{tr(G_{A}⊙G_{A})/n}$, the additive × dominance terms as $G_{AD}=\frac{G_{A}⊙G_{D}}{tr(G_{A}⊙G_{D})/n}$, and the dominance × dominance term as $G_{DD}=\frac{G_{D}⊙G_{D}}{tr(G_{D}⊙G_{D})/n}$.

#### 2.3.4. Model Including Additive Effects

The model used to perform additive PBLUP-A and GBLUP-A was:

(1) $$\begin{array}{r} y=X\beta +Z_{a}a+\varepsilon \end{array}$$

where ***y*** is the vector of environmental adjusted phenotypes, **β** is the vector of fixed effects (overall mean), ***a*** is the vector of additive genetic effects, and is assumed to follow a normal distribution with expectations of $\sim N\left(0,A\sigma_{a}^{2}\right)$ or $\sim N(0,G_{A}\sigma_{a}^{2})$ for pedigree- and genomic-based relationship matrices, respectively; **A** and **G_*A*_** were described above; $\sigma_{a}^{2}$ is the additive genetic variance and $\varepsilon \sim N\left(0,I\sigma_{\varepsilon }^{2}\right)$ is the vector of random residual effects, where **I** denotes the identity matrix and $\sigma_{\varepsilon }^{2}$ is the residual variance. **X** and **Z_*A*_** are the incidence matrices for **β** and ***a***, respectively.

#### 2.3.5. Model Including Dominance Effects

For the PBLUP-AD and GBLUP-AD models:

(2) $$\begin{array}{r} y=X\beta +Z_{a}a+Z_{d}d+\varepsilon \end{array}$$

where ***d*** is the vector of random dominance effects which follows a normal distribution with $\sim N\left(0,D\sigma_{d}^{2}\right)$ or $\sim N(0,G_{D}\sigma_{d}^{2})$, for the variance components using pedigree- or genomic-based relationship matrices, respectively;**D** and **G_*D*_** were described above; $\sigma_{d}^{2}$ is the dominance genetic variance and **Z_*d*_** is the incidence matrix for ***d***.

#### 2.3.6. Model Including Epistatic Effects

For the model including all effects (GBLUP-ADE):

(3) $$\begin{array}{r} y=X\beta +Z_{a}a+Z_{d}d+Z_{e1}e_{aa}+Z_{e2}e_{ad}+Z_{e3}e_{dd}+\varepsilon \end{array}$$

where ***e*_*aa*_**, ***e*_*ad*_**, and ***e*_*dd*_** are the vectors of random additive × additive, additive × dominance, and dominance × dominance epistatic effects; which are assumed to follow normal distributions with expectations $\sim N(0,G_{AA}\sigma_{aa}^{2})$, $\sim N(0,G_{AD}\sigma_{ad}^{2})$, and $\sim N(0,G_{DD}\sigma_{dd}^{2})$, respectively; and where $\sigma_{aa}^{2}$, $\sigma_{ad}^{2}$, and $\sigma_{dd}^{2}$ are the additive × additive, additive × dominance and dominance × dominance, epistatic interaction variances; **Z_*e*1_**, **Z_*e*2_**, and **Z_*e*3_** are the incidence matrices for ***e*_*aa*_**, ***e*_*ad*_**, and ***e*_*dd*_**, respectively.

### 2.4. Model Evaluation

The five models were compared based on the Akaike information criterion (AIC) for each trait. Based on the full dataset and depending on the model used, goodness-of-fit was evaluated by calculating the correlation between estimated total genetic or additive genetic values (respectively ${\hat{G}}_{full}$ and ${\hat{A}}_{full}$), and the adjusted phenotypes of individual trees (*y*_*full*_), i.e., $r\left({\hat{G}}_{full},y_{full}\right)$ and $r\left({\hat{A}}_{full},y_{full}\right)$. The significance of the correlation was examined using *t*-test. Additionally, standard error of the predictions (SEPs) was compared to evaluate the precision of the predicted estimated total genetic values (EGVs) for models containing nonadditive effects (i.e., PBLUP-AD, GBLUP-AD, and GBLUP-ADE) or estimated breeding values (EBVs) for models with only additive effects (i.e., PBLUP-A and GBLUP-A).

### 2.5. Heritabilities

For all models fitted, narrow-sense heritability was estimated as

(4) $$\begin{array}{r} {\hat{h}}^{2}=\frac{{\hat{\sigma }}_{a}^{2}}{{\hat{\sigma }}_{p}^{2}} \end{array}$$

Broad-sense heritability was estimated as

(5) $$\begin{array}{r} {\hat{H}}^{2}=\frac{{\hat{\sigma }}_{g}^{2}}{{\hat{\sigma }}_{p}^{2}} \end{array}$$

where ${\hat{\sigma }}_{g}^{2}$ is the estimated genetic variance predicted as ${\hat{\sigma }}_{a}^{2}+{\hat{\sigma }}_{d}^{2}$ for models with only additive and dominance effects (PBLUP-AD and GBLUP-AD); whereas, for the model with epistatic effects (GBLUP-ADE), it was estimated as ${\hat{\sigma }}_{g}^{2}={\hat{\sigma }}_{a}^{2}+{\hat{\sigma }}_{d}^{2}+{\hat{\sigma }}_{aa}^{2}+{\hat{\sigma }}_{ad}^{2}+{\hat{\sigma }}_{dd}^{2}$; ${\hat{\sigma }}_{p}^{2}$ denotes the predicted phenotypic variance and is the sum of the estimated total genetic and residual variances, and varied according to the model fitted.

### 2.6. Predictive Ability, Predictive Accuracy, and Cross-Validation

Ten-fold cross-validation with 10 replicates was used to estimate the predictive ability and predictive accuracy for all models. Estimations were performed within each fold and averaged across folds and replicates. The composition of the training and validation set was already evaluated in Calleja-Rodríguez et al. (2020).

The predictive ability (*r*_1_) was defined as the Pearson product-moment correlation between the cross-validated estimated total genetic (${\hat{G}}_{VP}$) or additive (${\hat{A}}_{VP}$) values and the adjusted phenotypes (*y*), i.e., $r_{1}=corr\left({\hat{G}}_{VP},y\right)$ or $r_{1}=corr\left({\hat{A}}_{VP},y\right)$; i.e., if the model predictions were only additive genetic effects, ${\hat{A}}_{VP}$ estimations were used, whereas for models containing both additive and nonadditive effects, *r*_1_ was estimated separately for ${\hat{G}}_{VP}$ and ${\hat{A}}_{VP}$.

The predictive accuracy (*r*_2_) was estimated according to Legarra et al. (2008), as *r*_1_ scaled by the square root of heritability, as $r_{2}=corr\left({\hat{A}}_{VP},y\right)/\hat{h}$, where $\hat{h}$ is the square root of narrow-sense heritability. Additionally, for the genetic effects models PBLUP-AD, GBLUP-AD, and GBLUP-ADE, *r*_2_ was also estimated as $r_{2}=corr\left({\hat{G}}_{VP},y\right)/\hat{H}$ where $\hat{H}$ is the square root of the broad-sense heritability.

In addition, for all models, Spearman's rank correlations between EGV and EBV rankings were also evaluated.

### 2.7. Expected Response of GS

The responses of genomic selection (RGS) and traditional phenotypic selection (RPS) were calculated as percentage of the average population following Resende et al. (2017),

(6) $$\begin{array}{r} RGS\left(\%\right)=RPS\left(\%\right)=\left(\frac{\bar{EGV_{S}}-\bar{EGV_{0}}}{\bar{EGV_{0}}}\right)\times 100 \end{array}$$

where $\bar{EGV_{S}}$ is the average of the expected genetic values for the individuals selected and estimated from PBLUP-AD, GBLUP-AD, GBLUP-AD, and GBLUP-ADE, and $\bar{EGV_{0}}$ is the average of genetic values of the population. When models only contain additive effects, i.e., GBLUP-A and PBLUP-A, $\bar{EGV_{S}}$ refers to the average of expected breeding values of the individuals selected and $\bar{EGV_{0}}$ is the average of breeding values of the population. The RGS is used for GBLUP models and RPS for PBLUP models.

Since one of the greatest advantages of GS in conifer breeding is the possibility to shorten the breeding cycle length, we compared the RGS per year (RGS/year) of GBLUP-A, GBLUP-AD, and GBLUP-ADE models, with the response of conventional phenotypic selection per year (RPS/year) of PBLUP-A and PBLUP-AD models, by assuming a reduction of 50% breeding cycle (11 years) in the GS scenario compared with the full cycle length (23 years) in the PS scenario.

### 2.8. Expected Genetic Gain

The expected genetic gains (Δ*G*) were estimated for two types of selection and deployment strategies, as a percentage of the overall mean ($\bar{y}$) and adapted from Mullin and Park (1992) and Chen et al. (2020). The strategies evaluated were (1) selection based on conventional testing for individual forward mass selection and grafted seed orchard (i.e., only additive effects), and (2) early GS with individual forward mass selection for vegetative propagation (i.e., total genetic effects).

The expected genetic for strategies 1 and 2 was estimated, respectively as,

(7) $$\begin{array}{r} \Delta G_{\hat{h^{2}}}=\frac{100{\hat{h}}^{2}i}{\bar{y}}\sqrt{{\hat{\sigma }}_{p}^{2}} \end{array}$$

(8) $$\begin{array}{r} \Delta G_{\hat{H^{2}}}=\frac{100{\hat{H}}^{2}i}{\bar{y}}\sqrt{{\hat{\sigma }}_{p}^{2}} \end{array}$$

where *i* is the selection intensity of *i* = 2.67 (i.e., 1% of the population selected).

## 3. Results

### 3.1. Model Evaluation

Generally, the lowest AICs were observed for PBLUP-A and PBLUP-AD models for almost all traits, but no considerable differences between the AIC values were obtained (Table 1).

**Table 1:** Summary of the genetic parameter estimations, including additive (${\hat{\sigma }}_{a}^{2}$), dominance (${\hat{\sigma }}_{d}^{2}$), epistatic additive × additive (${\hat{\sigma }}_{aa}^{2}$), epistatic additive × dominance (${\hat{\sigma }}_{ad}^{2}$), epistatic dominance × dominance (${\hat{\sigma }}_{dd}^{2}$), residual variances (${\hat{\sigma }}_{e}^{2}$), narrow- and broad-sense heritabilities (${\hat{h}}^{2}$ and ${\hat{H}}^{2}$, respectively), and Akaike information criterion (AIC) for each model and trait.

**Trait**	**Model**	**AIC**	**${\hat{\sigma }}_{a}^{2}$**	**${\hat{\sigma }}_{d}^{2}$**	**${\hat{\sigma }}_{aa}^{2}$**	**${\hat{\sigma }}_{ad}^{2}$**	**${\hat{\sigma }}_{dd}^{2}$**	**${\hat{\sigma }}_{e}^{2}$**	**${\hat{h}}^{2}$**	**${\hat{H}}^{2}$**
Ht1	PBLUP-A	5788.85	309.09 (118.88)	–	–	–	–	1469.49 (114.45)	0.17 (0.06)	–
	PBLUP-AD	5786.88	269.57 (122.53)	501.60 (283.39)	–	–	–	1009.78 (263.65)	0.15 (0.07)	0.43 (0.15)
	GBLUP-A	5793.20	328.42 (120.30)	–	–	–	–	1458.70 (117.30)	0.18 (0.06)	–
	GBLUP-AD	5795.20	328.42 (120.39)	0.00 (0.00)^*^	–	–	–	1458.70 (117.30)	0.18 (0.06)	0.18 (0.06)
	GBLUP-ADE	5801.07	299.03 (142.39)	0.00 (0.00)^*^	138.34 (380.26)	0.00 (0.00)^*^	0.00 (0.00)^*^	1359.88 (294.99)	0.17 (0.08)	0.24 (0.18)
Ht2	PBLUP-A	6875.41	3828.13 (1090.64)	–	–	–	–	5818.22 (726.37)	0.39 (0.09)	–
	PBLUP-AD	6876.84	3719.17 (1093.87)	847.19 (1193.23)	–	–	–	5068.48 (1254.57)	0.39 (0.10)	0.47 (0.14)
	GBLUP-A	6890.86	2999.85 (733.46)	–	–	–	–	6443.27 (584.16)	0.32 (0.07)	–
	GBLUP-AD	6892.76	2963.10 (744.24)	135.05 (557.15)	–	–	–	6343.47 (712.08)	0.31 (0.07)	0.33 (0.08)
	GBLUP-ADE	6895.17	2166.02 (819.39)	0.56 (536.44)	3573.72 (2090.50)	0.01 (0.00)^*^	0.01 (0.00)^*^	3936.31 (1502.41)	0.22 (0.08)	0.59 (0.16)
DBH1	PBLUP-A	4998.46	148.85 (49.45)	–	–	–	–	456.93 (40.62)	0.25 (0.07)	–
	PBLUP-AD	5000.46	148.85 (49.45)	0.00 (0.00)^*^	–	–	–	456.93 (40.62)	0.25 (0.07)	0.25 (0.07)
	GBLUP-A	5007.54	132.64 (40.75)	–	–	–	–	471.71 (38.19)	0.22 (0.06)	–
	GBLUP-AD	5009.55	132.64 (40.75)	0.00 (0.00)^*^	–	–	–	471.71 (38.19)	0.22 (0.06)	0.22 (0.06)
	GBLUP-ADE	5015.53	129.79 (48.38)	0.00 (0.00)^*^	13.76 (125.13)	0.00 (0.00)^*^	0.00 (0.00)^*^	461.83 (99.53)	0.21 (0.08)	0.24 (0.18)
DBH2	PBLUP-A	5216.79	161.69 (57.95)	–	–	–	–	625.69 (51.62)	0.21 (0.07)	–
	PBLUP-AD	5217.50	152.87 (59.02)	115.72 (111.27)	–	–	–	519.30 (109.33)	0.19 (0.07)	0.34 (0.14)
	GBLUP-A	5219.67	158.20 (40.75)	–	–	–	–	627.20 (49.879)	0.20 (0.06)	–
	GBLUP-AD	5221.43	151.11 (52.86)	28.06 (55.45)	–	–	–	606.24 (63.89)	0.19 (0.06)	0.23 (0.08)
	GBLUP-ADE	5225.99	107.21 (61.39)	7.22 (56.47)	239.73 (193.79)	0.00 (0.00)^*^	0.00 (0.00)^*^	449.86 (138.42)	0.13 (0.08)	0.44 (0.18)
MFA	PBLUP-A	2541.56	4.88 (1.51)	–	–	–	–	11.44 (1.14)	0.30 (0.08)	–
	PBLUP-AD	2541.56	4.88 (1.51)	0.00 (0.00)^*^	–	–	–	11.44 (1.14)	0.30 (0.08)	0.30 (0.08)
	GBLUP-A	2547.74	5.47 (1.39)	–	–	–	–	11.06 (1.07)	0.33 (0.07)	–
	GBLUP-AD	2549.74	5.47 (1.39)	0.00 (0.00)^*^	–	–	–	11.06 (1.07)	0.33 (0.07)	0.33 (0.07)
	GBLUP-ADE	2555.74	5.47 (1.39)	0.00 (0.00)^*^	0.00 (0.00)^*^	0.00 (0.00)^*^	0.00 (0.00)^*^	11.06 (1.07)	0.33 (0.07)	0.33 (0.07)
MOEs	PBLUP-A	1377.57	1.31 (0.37)	–	–	–	–	1.81 (0.24)	0.42 (0.10)	–
	PBLUP-AD	1379.57	1.31 (0.37)	0.00 (0.00)^*^	–	–	–	1.81 (0.24)	0.42 (0.10)	0.42 (0.10)
	GBLUP-A	1383.02	1.34 (0.28)	–	–	–	–	1.78 (0.19)	0.43 (0.07)	–
	GBLUP-AD	1385.02	1.34 (0.28)	0.00 (0.00)^*^	–	–	–	1.78 (0.19)	0.43 (0.07)	0.43 (0.07)
	GBLUP-ADE	1391.02	1.34 (0.28)	0.00 (0.00)^*^	0.00 (0.00)^*^	0.00 (0.00)^*^	0.00 (0.00)^*^	1.78 (0.19)	0.43 (0.07)	0.43 (0.07)
DEN	PBLUP-A	5233.81	407.20 (110.34)	–	–	–	–	497.16 (70.32)	0.45 (0.10)	–
	GBLUP-A	5232.72	376.82 (75.66)	–	–	–	–	506.02 (52.22)	0.43 (0.07)	–
	GBLUP-AD	5234.72	376.81 (75.66)	0.00 (0.00)^*^	–	–	–	506.03 (52.22)	0.43 (0.07)	0.43 (0.07)
	GBLUP-ADE	5239.82	341.84 (83.2)	0.00 (0.00)^*^	176.33 (183.68)	0.00 (0.00)^*^	0.00 (0.00)^*^	378.78 (138.75)	0.38 (0.08)	0.58 (0.17)
MOEd	PBLUP-A	932.29	0.81 (0.22)	–	–	–	–	0.86 (0.13)	0.48 (0.10)	–
	PBLUP-AD	934.29	0.81 (0.21)	0.00 (0.00)^*^	–	–	–	0.86 (0.13)	0.48 (0.10)	0.48 (0.10)
	GBLUP-A	946.17	0.68 (0.14)	–	–	–	–	0.96 (0.10)	0.42 (0.07)	–
	GBLUP-AD	948.17	0.68 (0.14)	0.00 (0.00)^*^	–	–	–	0.96 (0.10)	0.42 (0.07)	0.42 (0.07)
	GBLUP-ADE	951.17	0.54 (0.15)	0.00 (0.00)^*^	0.58 (0.35)	0.00 (0.00)^*^	0.00 (0.00)^*^	0.56 (0.25)	0.32 (0.08)	0.67 (0.16)

*Standard errors (SEs) in between parentheses*.

* *represents values fixed at the boundary in ASReml output files and considered as null*.

The highest goodness-of-fit was between total genetic values from the GBLUP-ADE model and adjusted phenotypes for all traits except Ht1, which had its highest goodness-of-fit for the total genetic values estimated from PBLUP-AD (Table 2). All models with nonadditive effects exhibited higher goodness-of-fit for the total genetic values when nonadditive effects were present. However, all models showed similar goodness-of-fit when additive genetic values $\left({\hat{A}}_{full}\right)$ were correlated with phenotypes (*y*_*full*_).

**Table 2:** Goodness-of-fit: correlation between phenotypes (*y*_*full*_) and additive genetic value (${\hat{A}}_{full}$) or total genetic value (${\hat{G}}_{full}$) of full data set, for each trait and each genomic or pedigree BLUP model.

**Trait**	**Genetic**	**GBLUP**		**PBLUP**	

	**effects**	$r\left({\hat{A}}_{\text{full}},y_{\text{full}}\right)$	$r\left({\hat{G}}_{\text{full}},y_{\text{full}}\right)$	$r\left({\hat{A}}_{\text{full}},y_{\text{full}}\right)$	$r\left({\hat{G}}_{\text{full}},y_{\text{full}}\right)$
Ht1	A	0.721	–	0.680	–
	AD	0.721	0.720	0.680	0.950
	ADE	0.721	0.830	–	–
Ht2	A	0.794	–	0.840	–
	AD	0.794	0.808	0.840	0.905
	ADE	0.783	0.966	–	–
DBH1	A	0.732	–	0.736	–
	AD	0.732	0.732	0.736	0.736
	ADE	0.732	0.763	–	–
DBH2	A	0.721	–	0.705	–
	AD	0.719	0.772	0.705	0.889
	ADE	0.712	0.947	–	–
MFA	A	0.813	–	0.776	–
	AD	0.813	0.813	0.776	0.776
	ADE	0.813	0.813	–	–
MOEs	A	0.860	–	0.853	–
	AD	0.860	0.860	0.853	0.853
	ADE	0.860	0.860	–	–
DEN	A	0.856	–	0.869	–
	AD	0.856	0.856	0.869	0.869
	ADE	0.852	0.945	–	–
MOEd	A	0.848	–	0.884	–
	AD	0.848	0.848	0.884	0.884
	ADE	0.838	0.973	–	–

*Correlations are statistically significant at 0.01*.

The comparison between SEPs for all models are presented in Table 3 and Supplementary Figures 1–6. The precision of EBVs and EGVs based on SEPs comparison between PBLUP models, revealed similar mean values of SEPs for most traits regardless of the PBLUP model used, except for Ht1, Ht2, and DBH2, that were slightly smaller for PBLUP-A. While, comparing GBLUP models, the lowest SEPs were produced by A and/or AD models. The mean SEPs values of EBVs from GBLUP-A were slightly lower than SEPs of EBVs from PBLUP-A for Ht2, DBH1, DEN, and MOEd. Yet, comparison of mean SEPs values of EBVs and EGVs for growth traits, DEN and MOEd, were somewhat smaller with GBLUP-A and GBLUP-AD compared to PBLUP-AD. GBLUP-ADE revealed the highest SEPs for all traits evaluated in the current study with the only exception of Ht1 for which the highest SEPs were observed with PBLUP-AD model. Standard deviations (SDs) of SEPs were highest for GBLUP models, irrespective of the genetic effects evaluated.

**Table 3:** Basic descriptive statistics estimated for SEPs (SE Error of Predictions) of each trait, and pedigree- and genomic-based models.

**Trait**	**Statistic**	**PBLUP-A**	**PBLUP-AD**	**GBLUP-A**	**GBLUP-AD**	**GBLUP-ADE**
Ht1	$\bar{SEPs}$	13.18	19.96	14.38	14.38	16.47
	*max*_*SEPs*_	16.17	20.98	19.33	19.33	22.87
	*min*_*SEPs*_	13.38	19.60	12.56	12.56	13.60
	*SD*_*SEPs*_	0.51	0.27	0.82	0.82	1.02
Ht2	$\bar{SEPs}$	40.92	43.82	38.86	39.51	45.62
	*max*_*SEPs*_	48.36	54.33	50.96	52.51	81.73
	*min*_*SEPs*_	40.02	43.01	34.98	35.51	38.88
	*SD*_*SEPs*_	1.53	1.18	2.25	2.26	2.29
DBH1	$\bar{SEPs}$	9.03	9.03	8.87	8.87	9.22
	*max*_*SEPs*_	12.35	12.35	11.85	11.85	12.47
	*min*_*SEPs*_	8.77	8.77	7.82	7.82	7.98
	*SD*_*SEPs*_	0.39	0.39	0.52	0.52	0.55
DBH2	$\bar{SEPs}$	9.71	12.32	9.83	10.50	13.24
	*max*_*SEPs*_	11.45	14.47	13.18	14.01	17.58
	*min*_*SEPs*_	9.43	12.06	8.62	9.20	10.68
	*SD*_*SEPs*_	0.37	0.25	0.57	0.61	0.72
MFA	$\bar{SEPs}$	1.57	1.57	1.64	1.64	1.64
	*max*_*SEPs*_	1.86	1.86	2.41	2.41	2.41
	*min*_*SEPs*_	1.53	1.53	1.48	1.48	1.48
	*SD*_*SEPs*_	0.06	0.06	0.10	0.10	0.10
MOEs	$\bar{SEPs}$	0.74	0.74	0.75	0.75	0.75
	*max*_*SEPs*_	0.88	0.88	1.18	1.18	1.18
	*min*_*SEPs*_	0.73	0.73	0.67	0.67	0.67
	*SD*_*SEPs*_	0.03	0.03	0.04	0.04	0.04
DEN	$\bar{SEPs}$	12.82	12.82	12.59	12.59	13.55
	*max*_*SEPs*_	15.39	15.39	19.70	19.70	25.33
	*min*_*SEPs*_	12.56	12.56	11.23	11.23	11.97
	*SD*_*SEPs*_	0.47	0.47	0.73	0.73	0.74
MOEd	$\bar{SEPs}$	0.56	0.56	0.54	0.54	0.58
	*max*_*SEPs*_	0.68	0.68	0.84	0.84	1.23
	*min*_*SEPs*_	0.55	0.55	0.48	0.48	0.51
	*SD*_*SEPs*_	0.02	0.02	0.03	0.03	0.03

*$\bar{SEPs}$, max_SEPs_, min_SEPs_ and SD_SEPs_ respectively represent mean, max, min, and standard deviation (SD) values of SEPs*.

### 3.2. Variance Components and Heritability Estimates

Estimates of ${\hat{h}}^{2}$ ranged from low to moderate (0.15–0.48), being higher with PBLUP for Ht2, DBH1, DEN, and MOEd, while for the remaining traits, ${\hat{h}}^{2}$ were similar or slightly higher with GBLUP models (Table 1). Usually, wood quality traits had higher ${\hat{h}}^{2}$ than growth traits.

Based on nonadditive models, ${\hat{H}}^{2}$ was moderate (0.23–0.67) with some indication of higher estimates with GBLUP compared to PBLUP. Indeed, the highest estimates of ${\hat{H}}^{2}$ were obtained by the model with epistatic effects (i.e., GBLUP-ADE) for Ht2 (0.59), DEN (0.58), and MOEd (0.67), whereas low or moderate estimated ${\hat{H}}^{2}$ were observed for the remaining traits.

**Figure 1:**
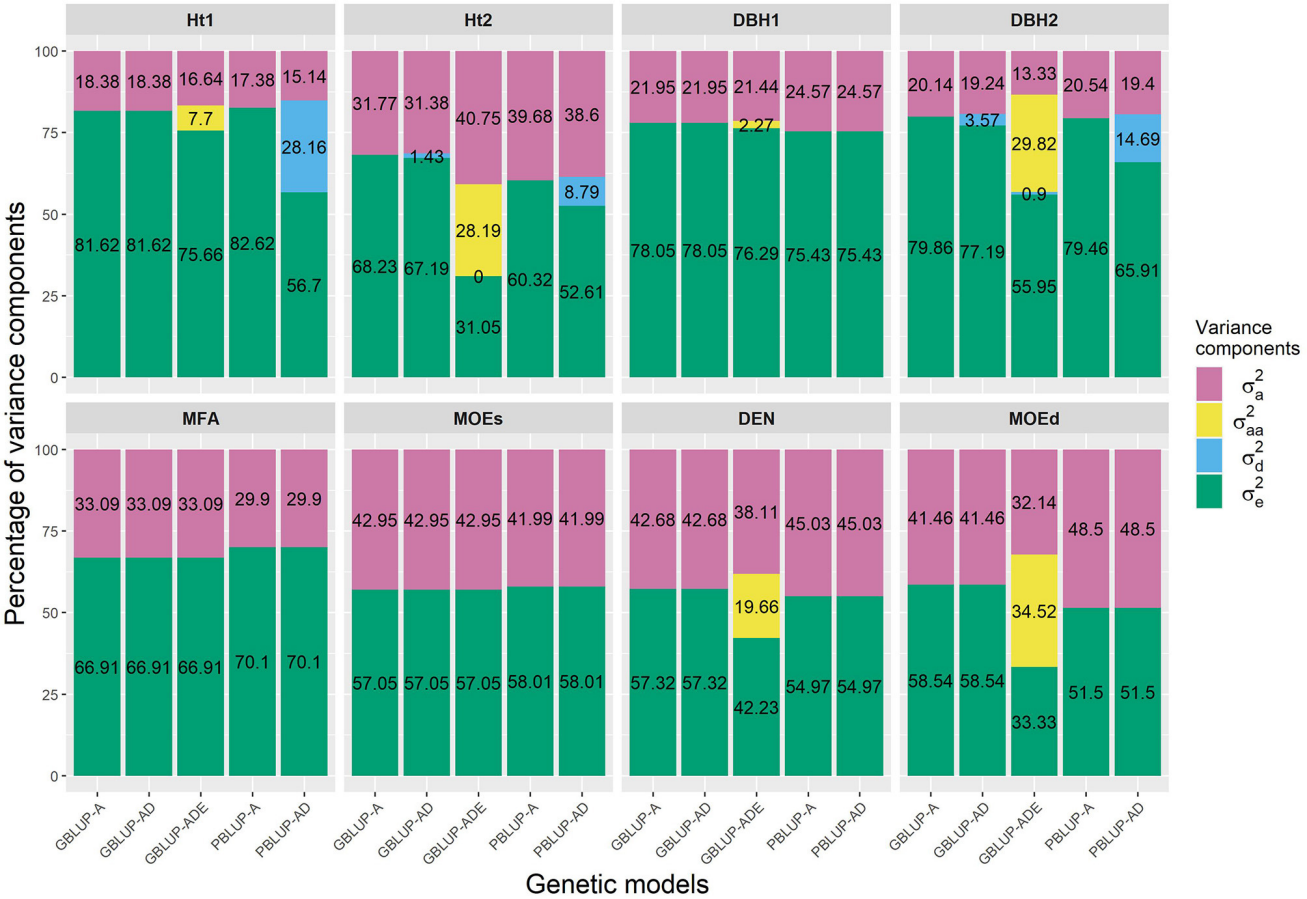
Percentages of the different variance components for each genomic- and pedigree-based BLUP model and trait. $\sigma_{a}^{2}$, $\sigma_{aa}^{2}$, $\sigma_{d}^{2}$, and $\sigma_{e}^{2}$ denotes additive, epistatic additive × additive, dominance, and residual variances, respectively.

The PBLUP-AD model estimated a considerable amount of dominance variance particularly for Ht1 and DBH2, accounting, respectively, for 28.16 and 14.69% of the total variance (Figure 1 and Table 1). Nevertheless, by using GBLUP-AD, the dominance variance for these three traits shrunk and even more when the epistatic effects were included in the model (Figure 1). Additive genetic and residual variance components decreased for GBLUP when estimating epistatic effects for growth, DEN and MOEd. For DEN, MOEd, and all growth traits, the epistatic variance was observed but their percentages of the total variance depended on the trait, being lower at the first assessment of Ht and DBH. No epistatic variance, due to additive × dominance or dominance × dominance interactions, was determined for any of the traits. There were two wood stiffness traits (MFA and MOEs) that did not show any nonadditive genetic variation at all.

### 3.3. Predictive Ability, Predictive Accuracy, and Spearman Rank Correlations

**Figure 2:**
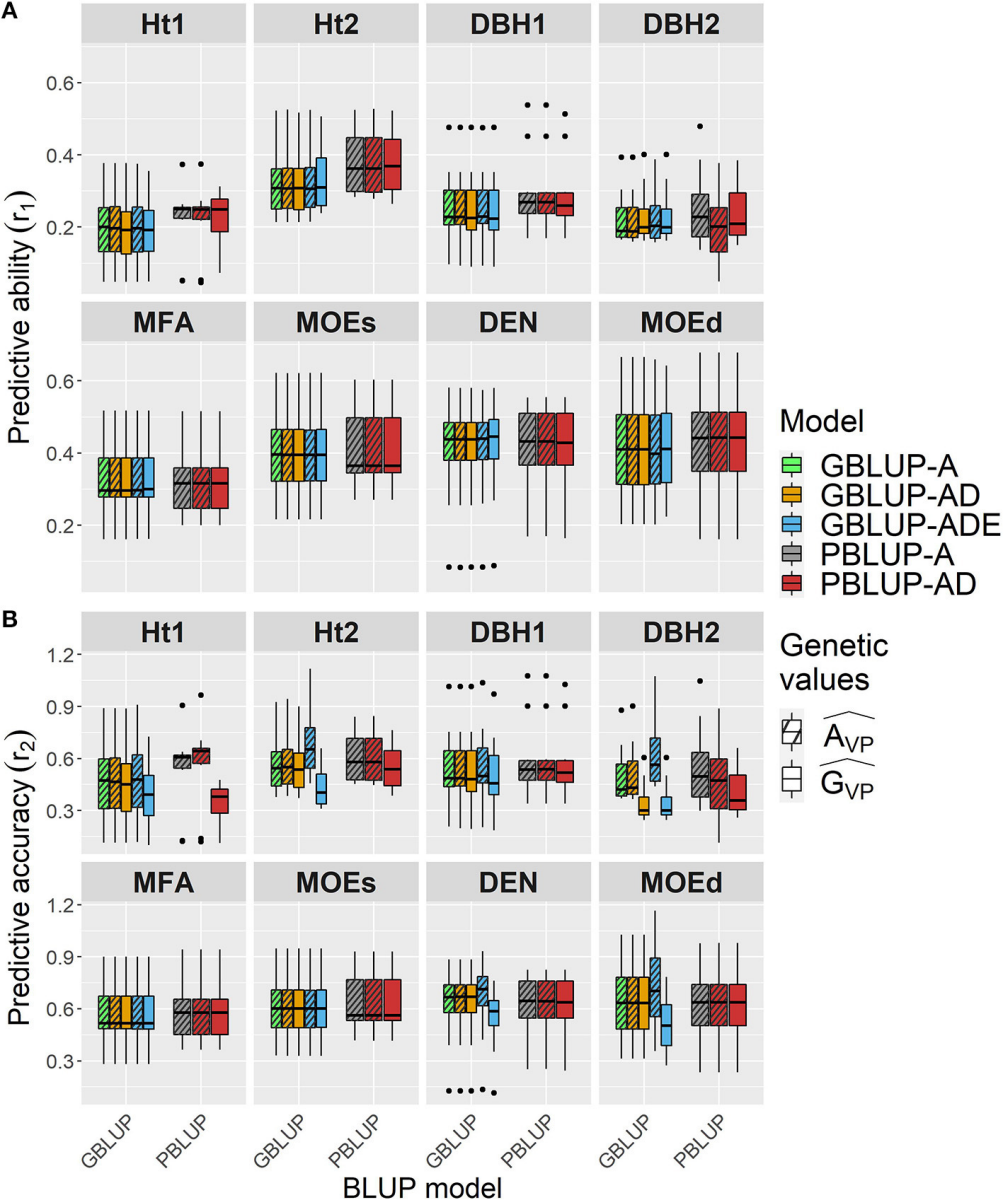
Boxplots of the **(A)** predictive abilities (*r*_1_) and **(B)** predictive accuracies (*r*_2_) assessed for all traits and genomic- and pedigree-based models with cross-validated estimated total genetic (${\hat{G}}_{VP}$) or additive (${\hat{A}}_{VP}$) values.

Wood traits showed higher *r*_1_ than growth traits, and they were similar among all models, whatsoever genetic effects were considered in each model, apart from Ht2 and DBH1 for which *r*_1_ was slightly higher for both PBLUP models (Figure 2A and Table 4). For Ht2, DBH1, and DBH2, *r*_1_ was to some extent lower for total genetic values compared with additive genetic values when estimated with PBLUP.

**Table 4:** Predictive ability (*r*_1_) and predictive accuracy (*r*_2_) estimated with total genetic values of the validation population (${\hat{G}}_{VP}$) and additive genetic values of the validation population (${\hat{A}}_{VP}$) for all pedigree and genomic BLUP models and traits.

**Trait**	**Genetic**	**GBLUP**				**PBLUP**			
	**effects**	$r_{1}\left({\hat{A}}_{VP},y\right)$	$r_{1}\left({\hat{G}}_{VP},y\right)$	$r_{2}\left({\hat{A}}_{VP},y\right)$	$r_{2}\left({\hat{G}}_{VP},y\right)$	$r_{1}\left({\hat{A}}_{VP},y\right)$	$r_{1}\left({\hat{G}}_{VP},y\right)$	$r_{2}\left({\hat{A}}_{VP},y\right)$	$r_{2}\left({\hat{G}}_{VP},y\right)$
Ht1	A	0.20 (0.03)	–	0.46 (0.08)	–	0.22 (0.03)	–	0.53 (0.08)	–
	AD	0.20 (0.03)	0.19 (0.03)	0.46 (0.08)	0.44(0.08)	0.22 (0.03)	0.22 (0.03)	0.57 (0.08)	0.54 (0.04)
	ADE	0.20 (0.03)	0.19 (0.03)	0.47 (0.08)	0.38 (0.06)	–	–	–	–
Ht2	A	0.33 (0.03)	–	0.59 (0.06)	–	0.38 (0.03)	–	0.61 (0.05)	–
	AD	0.33 (0.03)	0.33 (0.03)	0.60 (0.06)	0.58 (0.06)	0.38 (0.03)	0.37(0.03)	0.61 (0.05)	0.55 (0.04)
	ADE	0.33 (0.03)	0.34 (0.03)	0.71 (0.07)	0.44 (0.04)	–	–	–	–
DBH1	A	0.26 (0.03)	–	0.54 (0.07)	–	0.30 (0.04)	–	0.58 (0.07)	–
	AD	0.26 (0.03)	0.25 (0.03)	0.54 (0.07)	0.54 (0.07)	0.30 (0.04)	0.29 (0.03)	0.59 (0.07)	0.58 (0.07)
	ADE	0.26 (0.03)	0.25 (0.03)	0.56 (0.07)	0.52 (0.07)	–	–	–	–
DBH2	A	0.23 (0.02)	–	0.50 (0.05)	–	0.25 (0.04)	–	0.55 (0.08)	–
	AD	0.23 (0.02)	0.22 (0.02)	0.52 (0.06)	0.46 (0.05)	0.25 (0.04)	0.24 (0.03)	0.58 (0.08)	0.42 (0.05)
	ADE	0.23 (0.04)	0.23 (0.04)	0.63 (0.07)	0.35 (0.04)	–	–	–	–
MFA	A	0.32 (0.04)	–	0.56 (0.06)	–	0.33 (0.04)	–	0.60 (0.06)	–
	AD	0.32 (0.04)	0.32 (0.04)	0.56 (0.06)	0.56 (0.06)	0.33 (0.04)	0.33 (0.04)	0.60 (0.06)	0.60 (0.06)
	ADE	0.32 (0.04)	0.32 (0.04)	0.56 (0.06)	0.56 (0.06)	–	–	–	–
MOEs	A	0.40 (0.04)	–	0.62 (0.06)	–	0.41 (0.04)	–	0.63 (0.06)	–
	AD	0.40 (0.04)	0.40 (0.04)	0.62 (0.06)	0.62 (0.06)	0.41 (0.04)	0.41 (0.04)	0.63 (0.06)	0.63 (0.06)
	ADE	0.40 (0.04)	0.40 (0.04)	0.62 (0.06)	0.62 (0.06)	–	–	–	–
DEN	A	0.40 (0.05)	–	0.62 (0.07)	–	0.41 (0.04)	–	0.61 (0.06)	–
	AD	0.40 (0.05)	0.40 (0.05)	0.62 (0.07)	0.62 (0.07)	0.41 (0.04)	0.41 (0.04)	0.61 (0.06)	0.61 (0.06)
	ADE	0.40 (0.04)	0.41 (0.05)	0.66 (0.07)	0.54 (0.06)	–	–	–	–
MOEd	A	0.42 (0.05)	–	0.65 (0.08)	–	0.43 (0.05)	–	0.63 (0.07)	–
	AD	0.42 (0.05)	0.42 (0.05)	0.65 (0.08)	0.65 (0.08)	0.43 (0.05)	0.43 (0.05)	0.63 (0.07)	0.63 (0.07)
	ADE	0.42 (0.05)	0.42 (0.05)	0.74 (0.09)	0.52 (0.06)	–	–	–	–

*Standard errors (SEs). A, AD, and ADE are respectively the acronyms of additive, additive + dominance, and additive + dominance + epistatic effects*.

Conversely, *r*_2_ varied depending on the genetic effects included in the GBLUP or PBLUP model for some traits (Figure 2B and Table 4). Using additive genetic effects estimated from GBLUP-ADE showed the highest *r*_2_ for Ht2, DBH2, DEN, and MOEd, whereas for the total genetic effects, this model exhibited the lowest values for almost all traits. In the cases that dominance effects were present and genetic values were used to estimate *r*_2_, both GBLUP-AD and PBLUP-AD produced similar values.

In general, *r*_1_ was similar among all models regardless of the genetic effects considered, whereas *r*_2_ was higher for additive genetic effects of growth traits than for the total genetic effects model used, being slightly higher when GBLUP-ADE was used for Ht2, DBH2, DEN, and MOEd.

Spearman ranking correlations revealed generally high correlations between EBVs or EGVs from GBLUP and PBLUP models (0.845–0.876), being even higher between both PBLUP models (0.998–1.000) and between the three GBLUP models (0.994–1.000; Table 5).

**Table 5:** Spearman's rank correlations between estimated total genetic values (EGV) and additive genetic values (EBV), for each trait and genomic- or pedigree-based BLUP model.

	**Ht1**					**Ht2**			

	**PBLUP-AD**	**GBLUP-A**	**GBLUP-AD**	**GBLUP-ADE**		**PBLUP-AD**	**GBLUP-A**	**GBLUP-AD**	**GBLUP-ADE**
PBLUP-A	0.998	0.856	0.857	0.855		1.000	0.875	0.876	0.869
PBLUP-AD		0.854	0.854	0.853			0.875	0.876	0.869
GBLUP-A			1.000	0.998				1.000	0.995
GBLUP-AD				0.998					0.995
	**DBH1**					**DBH2**			
	**PBLUP-AD**	**GBLUP-A**	**GBLUP-AD**	**GBLUP-ADE**		**PBLUP-AD**	**GBLUP-A**	**GBLUP-AD**	**GBLUP-ADE**
PBLUP-A	1.000	0.869	0.868	0.867		0.999	0.855	0.854	0.847
PBLUP-AD		0.869	0.868	0.867			0.853	0.853	0.846
GBLUP-A			1.000	0.999				0.999	0.993
GBLUP-AD				0.999					0.994
	**MFA**					**MOEs**			
	**PBLUP-AD**	**GBLUP-A**	**GBLUP-AD**	**GBLUP-ADE**		**PBLUP-AD**	**GBLUP-A**	**GBLUP-AD**	**GBLUP-ADE**
PBLUP A	1.000	0.851	0.851	0.852		1.000	0.845	0.845	0.845
PBLUP AD		0.851	0.851	0.852			0.845	0.845	0.845
GBLUP A			1.000	1.000				1.000	1.000
GBLUP AD				1.000					1.000
	**DEN**					**MOEd**			
	**PBLUP-AD**	**GBLUP-A**	**GBLUP-AD**	**GBLUP-ADE**		**PBLUP-AD**	**GBLUP-A**	**GBLUP-AD**	**GBLUP-ADE**
PBLUP-A	1.000	0.861	0.861	0.859		1.000	0.864	0.864	0.856
PBLUP-AD		0.861	0.861	0.859			0.864	0.864	0.856
GBLUP-A			1.000	0.999				1.000	0.997
GBLUP-AD				0.999					0.997

### 3.4. Expected Response of GS

**Figure 3:**
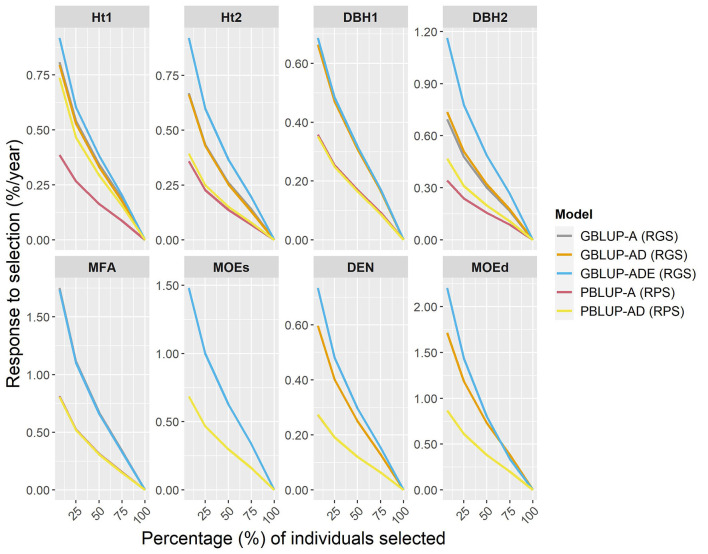
Percentages of the response of genomic selection (RGS) and conventional phenotypic selection (RPS) per year, for genomic- and pedigree-based models for all traits and different proportions of individuals selected (7, 25, 50, 75, and 100% individuals).

The expected percentage response of GS per year (RGS/year) was compared with the response to phenotypic selection per year (RPS/year), by assuming that the Scots pine breeding cycle could be shortened by 50% (from 23 to 11 years) by reducing field test periods and by stimulating early female flowering. To estimate RGS and RPS per year, different proportions of individuals were selected (Figure 3). The results showed that RGS/year was considerably higher than RPS/year for all traits, i.e., a relatively higher percentage of response to selection were obtained with genomic-based models for all traits and they were particularly noteworthy for wood traits. Indeed, for the top 7% of individuals (50 selected individuals), the RGS expected for wood traits ranged between 0.32 and 1.33% higher per year than with PBLUP models. The RGS/year for growth traits was between 0.26 and 0.82% higher than RPS/year, except for PBLUP-AD that was only 0.05% lower than GBLUP-AD, for Ht2. Generally, the GBLUP-ADE presented the largest RGS/year, while RPS/year for PBLUP models, was lower in all cases.

### 3.5. Expected Genetic Gain

Expected genetic gains were estimated for traits that showed substantial nonadditive genetic effects in the GBLUP model, i.e., growth traits, DEN, and MOEd. For the first deployment strategy based on conventional testing, results from the PBLUP model with additive effects were used, whereas for the second strategy, i.e., early GS, results from GBLUP-ADE model were used to account for the total genetic effects. A common selection intensity of 1% was used for both strategies. Expected genetic gains ($\Delta G_{{\hat{h}}^{2}}$) for the first deployment strategy, based on only additive effects, varied from 8.1 to 25.1%, and for the early GS strategy, based on total genetic effects, $\Delta G_{{\hat{H}}^{2}}$ ranged from 9.0 to 35.1% (Table 6). Thus, expected genetic gains were higher for all traits with the genomic deployment strategy, with the only exception of DBH1. However, for DBH1 ${\hat{h}}^{2}$ estimated from PBLUP model was higher than ${\hat{H}}^{2}$ obtained with GBLUP-ADE model (Table 1), which resulted in slightly higher genetic gains (9.4%) from conventional pedigree selection than early GS (9.0%) for this specific trait.

**Table 6:** Expected genetic gains (*%ΔG*), for growth traits (Ht1, Ht2, DBH1, and DBH2) and two different deployment strategies, with a selection intensity of *i* = 2.67 (1% of the population selected).

**%ΔG**	**Ht1**	**Ht2**	**DBH1**	**DBH2**	**DEN**	**MOEd**
$\Delta G_{\hat{h^{2}}}$	8.6	9.0	9.4	8.6	8.1	25.1
$\Delta G_{\hat{H^{2}}}$	12.2	13.6	9.0	18.1	10.4	35.1

## 4. Discussion

One of the greatest benefits of GS is the possibility of reducing the breeding cycle length with the consequent increase of genetic gain per year, but an additional advantage is the possibility to decompose the genetic variance into additive and nonadditive (both dominance and epistatic) effects more precisely without performing specific mating designs to generate full-sib families for clonal selection (El-Dien et al., 2016; Grattapaglia et al., 2018; Lebedev et al., 2020).

### 4.1. Model Fit With Additive and Nonadditive Effects

Based on AIC, GBLUP models performed better than PBLUP models in eucalypt hybrids (Tan et al., 2018), however in loblolly pine and Norway spruce (Chen et al., 2019) no obvious superiority of any model based on differences in AIC could be observed. On the contrary for Scots pine, AIC slightly increased when dominance and epistatic variance were accounted for in the models, similarly to the results reported for white spruce (El-Dien et al., 2016).

Our genomic marker-based models with the epistatic effects generally resulted in the highest correlations between the phenotypes and total genetic values (goodness-of-fit). Similar results were described in eucalypt hybrids (Bouvet et al., 2016; Tan et al., 2018) or simulation studies (Nazarian and Gezan, 2016), suggesting that total genetic values (i.e., including nonadditive effects) were more similar to phenotypes than only breeding values.

In the current study, PBLUP-A was only clearly advantageous for growth traits, and PBLUP-AD and GBLUP-ADE showed the highest SEPs. Hence, GBLUP-A and GBLUP-AD models produced good estimates of genetic values and breeding values, especially for wood traits. Two studies in interior spruce, *Picea glauca* (Moench) Voss × *Picea engelmannii* Parry ex Engelm., (El-Dien et al., 2018) and white spruce (El-Dien et al., 2016) reported the clear superiority of GBLUP models compared with PBLUP, based on the SEPs of breeding values, exhibiting the GBLUP-ADE model the lowest SEPs. Similar results were observed in loblolly pine (Munoz et al., 2014), but in Norway spruce (Chen et al., 2019) GBLUP models were distinctly better for wood traits.

### 4.2. Variance Components and Heritability

Narrow-sense heritability estimates in the current study were alike to previous studies in pines, generally higher for wood quality than growth traits (Haapanen et al., 1997; Fries and Ericsson, 2006; Baltunis et al., 2010). PBLUP model with only additive effects resulted in prominent higher estimates of narrow-sense heritability than GBLUP for height at 30 years old and MOEd, but very similar or superior with GBLUP models for the remaining traits evaluated in the current study. This could be explained by the genomic relationship matrix accounting for Mendelian sampling as based on realized genetic covariances between individuals that are identical by descent or by state (Visscher et al., 2006; VanRaden, 2008; Hayes et al., 2009). Either inflation or deflation of heritability estimates using PBLUP has been reported in earlier studies for other conifer species (El-Dien et al., 2015; Lenz et al., 2017; Chen et al., 2019). Increased accuracy of genetic parameters is well studied in genomic marker-based evaluations (Hayes et al., 2009; Resende et al., 2012; Resende Jr et al., 2012; El-Dien et al., 2018) which will lead to the more precise selection and increased genetic gains. Overestimation of narrow-sense heritability can be a consequence of inadequate estimation power of pedigree-based methods in full-sib families due to confounding of environmental effects (Baltunis et al., 2007) and nonadditive genetic effects confounded with additive genetic and nongenetic effects (Lee et al., 2010) and with epistatic effects (Munoz et al., 2014; Tan et al., 2018).

We could observe that growth traits were the only traits presenting dominance variation. When models also could account for a considerable epistatic variance, the dominance variance reduced along with the additive and residual variance components, thus lower narrow-sense heritabilities and higher broad-sense heritabilities were reached. For the traits not exhibiting any dominance effects, GBLUP-AD resulted in equal estimates of heritability as GBLUP-A but that may change by increasing the number of genotypes per family (El-Dien et al., 2016). Similar indications of dominance variation contributing to growth have been reported earlier with pedigree-based methods in a full-sib population of loblolly pine (Isik et al., 2003; Baltunis et al., 2007).

Interestingly, two wood quality traits, DEN and MOEd showed epistatic variance using GBLUP. This resulted in the increased estimates of broad-sense heritabilities and reduced additive and dominance variance components compared to the dominance model. Epistatic effects explaining an important part of the variation have been noticed for different traits in other tree species (Bouvet et al., 2016; Tan et al., 2018). In Scots pine, these are the first results indicating that not only dominance, but also epistatic effects may have a role in contributing to the variation. Similar effects of decreasing additive genetic variance when using genomic marker-based methods to estimate nonadditive genetic effects have been observed in loblolly pine (Munoz et al., 2014). Similar to our results, in an open-pollinated white spruce population, genomic-marker based model including epistatic effects, was able to distinguish the variance component more effectively, accounting for a considerable amount of variation for height and wood density, whereas pedigree-based models resulted in overestimated additive genetic variances as being confounded mainly with additive × additive epistatic effects (El-Dien et al., 2016). Also in eucalypts hybrids, adding the dominance and epistatic effects had a decreasing effect in both additive and dominance variances (Bouvet et al., 2016).

Despite the low number of individuals per family in the current study, we were able to estimate non-null epistatic variances. However, it may be expected that increasing the number of individuals per family would result in further improved estimates of nonadditive effects (Chen et al., 2020). In our study, the nature of epistatic variation was only type additive × additive for all traits which can be an indication of more information required to discern between the epistatic components. Epistatic variance falling into one component only has been observed earlier as well (Munoz et al., 2014; El-Dien et al., 2016, 2018).

### 4.3. Predictive Ability, Predictive Accuracy, and Spearman Rank Correlations

In forest tree breeding studies, accuracies of genomic models had been estimated with four methods, for example, predictive ability (*r*_1_) in Norway spruce (Chen et al., 2018) or eucaltyp hybrids (Tan et al., 2017); predictive accuracy (*r*_2_) in radiata pine (Klápšte et al., 2020) or *Populus nigra* L. (Pégard et al., 2020); theoretical accuracy (*r*_3_) in *Eucalyptus nitens* (Suontama et al., 2018) or white spruce (Lenz et al., 2020), and prediction accuracy (*r*_4_) in *Pseudotsuga menziesii* (Mirb.) Franco (Thistlethwaite et al., 2017), *Pinus pinaster* Ait. (Bartholomé et al., 2016) or *Pinus contorta* Dougl. ex. Loud. (Ukrainetz and Mansfield, 2020). Given that the correlation between an individual phenotype and its true breeding value cannot be larger than the square root of heritability, *r*_2_ is recognized as an unbiased estimation of accuracy of selection from n-fold-cross validation (Legarra et al., 2008; Meuwissen et al., 2013), thus *r*_2_ has begun to be more standard used in forest tree GS studies (Lenz et al., 2019, 2020; Calleja-Rodríguez et al., 2020; Klápšte et al., 2020; Pégard et al., 2020; Zhou et al., 2020).

Similar *r*_1_ were observed, for all models and traits evaluated for Scots pine, which agrees with previous studies (Bouvet et al., 2016; de Almeida Filho et al., 2016; Chen et al., 2019), but differs from other eucalyptus reports in which the genomic models performed better (Tan et al., 2018), yet in the same study inconsistencies in the original pedigree were detected, and a pseudo-pedigree was used. In a black poplar study, *r*_2_ did not improve when dominance effects were added to the additive effect models (Pégard et al., 2020), which is congruent with our results. However, in our case, the genomic model with epistatic effects performed better to predict additive values for growth and wood quality traits. These results suggest that considering epistatic variation in full-sib families in this population, and especially for some of the traits will result in better estimations of total genetic values.

Spearman's rank correlations may have amplified the benefits of genomic models in black poplar (Pégard et al., 2020) whereas in Norway spruce they were similar between pedigree-based and genomic-based models (Chen et al., 2019), coinciding with our results.

### 4.4. Expected Response of GS and Genetic Gains

Higher response of GS was detected as the ratio of individuals selected decrease in eucalypt hybrids (Resende et al., 2017) or Norway spruce (Chen et al., 2019). Congruent with those studies the same pattern was noticed, and a higher response was observed for GS models compared with traditional phenotypic selection, increasing proportionally to the ratio of individuals selected reaching the maximum when the best 50 individuals were selected. The reduction of 50% in the breeding cycle length assumed for Scots pine in the present study will only be possible by combining it with greenhouse flowering stimulation that will aid to produce female flowers at earlier ages (Almqvist, 2018; Calleja-Rodríguez et al., 2020), than the current flowering age for the species between 15 and 18 years of age (Matyas et al., 2004).

The GBLUP epistatic model outperformed the PBLUP and GBLUP additive and dominance models, in genomic response estimations for almost all traits. This increased response to selection was particularly striking for wood quality traits that did not show dominance effects neither with pedigree-based nor genomic-marker-based models. It can be considered something that should be further investigated in Scots pine with more genotyping and phenotyping for wood properties to get more confidence whether epistatic variation play an important role in wood quality traits.

Genetic gains are expected to increase with the use of GS by shortening breeding cycle lengths, increasing selection intensities, and estimating more accurate breeding or genetic values . To date, the Scots pine breeding program in Sweden comprises conventional progeny testing and breeding value prediction based on pedigree information (Rosvall, 2011) for which decomposition of variance in additive and nonadditive effects is not possible in most cases due to a limited number of full-sib or clonal individuals to evaluate. In the current study, expected genetic gains from conventional progeny testing were lower than those from GS, which accounted for the total genetic variance, unveiling that clonal deployment could be a desirable alternative for the species.

## 5. Conclusions

Pedigree-based models (PBLUP) resulted in the overestimation of additive genetic and dominance variance and larger residual error variances for most of the growth traits, wood density, and dynamic modulus of elasticity, compared to genomic-based models (GBLUP). For the GBLUP model with epistatic effects, only type additive × additive variance was noticed for growth traits, wood density, and dynamic modulus of elasticity. This partition of variance components decreased estimates of narrow-sense heritability, but higher broad-sense heritabilities were reached compared to the models with dominance effects. Dominance variation was not observed for wood quality traits. These results indicate that nonadditive genetic effects may have an important role in the variation of objective traits in the Swedish Scots pine breeding program and that the GS model would be more able to detect nonadditive genetic variance. GBLUP model with the epistatic effects exhibited the higher predictive ability of additive values and outperformed other models in terms of correlations between phenotypes and total estimated genetic values, which was especially noticeable for height at age 30, DBH at age 36, wood density, and modulus of elasticity. Future studies with more extensive phenotypic data and further developed genotyping platform would extend our knowledge on the role of nonadditive genetic effects in the breeding of Scots pine.

## Data Availability Statement

The data presented in the study are deposited in the NCBI SRA Bioproject repository, accession number PRJNA732895, and in the article Supplementary Material.

## Author Contributions

AC-R performed the data analysis, drafted the manuscript, participated in field sampling, performed DNA extractions, and obtained funding for open-access publishing. ZC participated in field sampling, performed the imputation of marker data, and assisted with script model development and editing of the final manuscript. MS assisted in drafting the manuscript and discussed the analysis. JP performed the SNP filtering and calling. HW designed the study, discussed the analyses, assisted in editing the final manuscript, and obtained funding. All authors contributed to the article and approved the submitted version.

## Conflict of Interest

The authors declare that the research was conducted in the absence of any commercial or financial relationships that could be construed as a potential conflict of interest.
