# Supplementary material for: Genomic Predictions With Nonadditive Effects Improved Estimates of Additive Effects and Predictions of Total Genetic Values in Pinus sylvestris
Source: Front Plant Sci. 2021 Jul 7;12:666820. doi: 10.3389/fpls.2021.666820 (PMC8294091; doi:10.3389/fpls.2021.666820)
Supplement: Supplementary file 1 [file Presentation_1.pdf]

Supplementary Material:

Genomic predictions with non-additive effects improved estimates of additive effects and predictions of total genetic values in *Pinus sylvestris*

1 SUPPLEMENTARY DATA

The raw phenotypic data and pedigree information can be found as Supplementary Data file Table 1.xlsx

2 SUPPLEMENTARY FIGURES

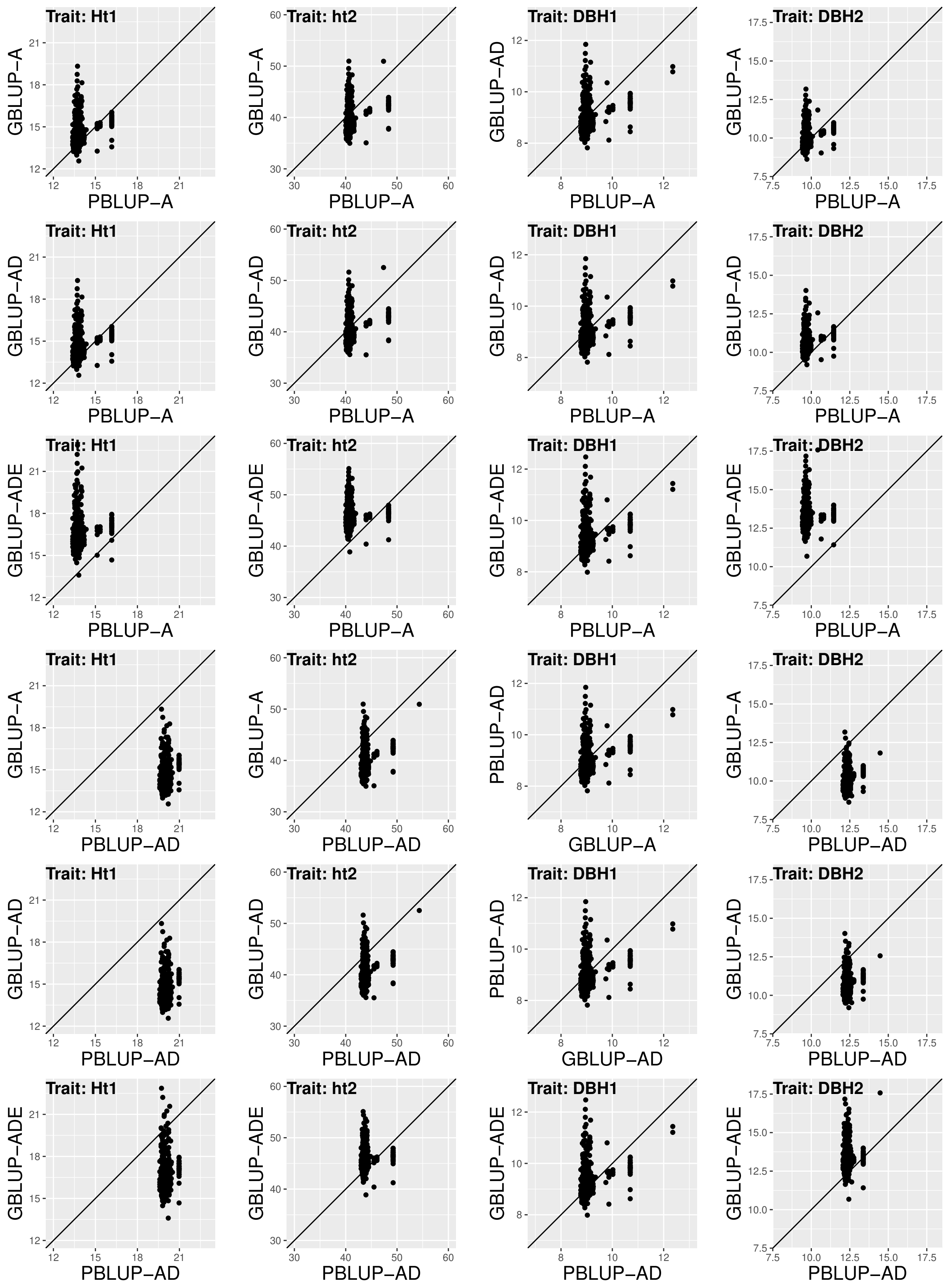

Figure S1. Comparison of Standard Error of Predictions (SEPs) of the estimated breeding- and genetic-values between GBLUP and PBLUP models, for growth traits.

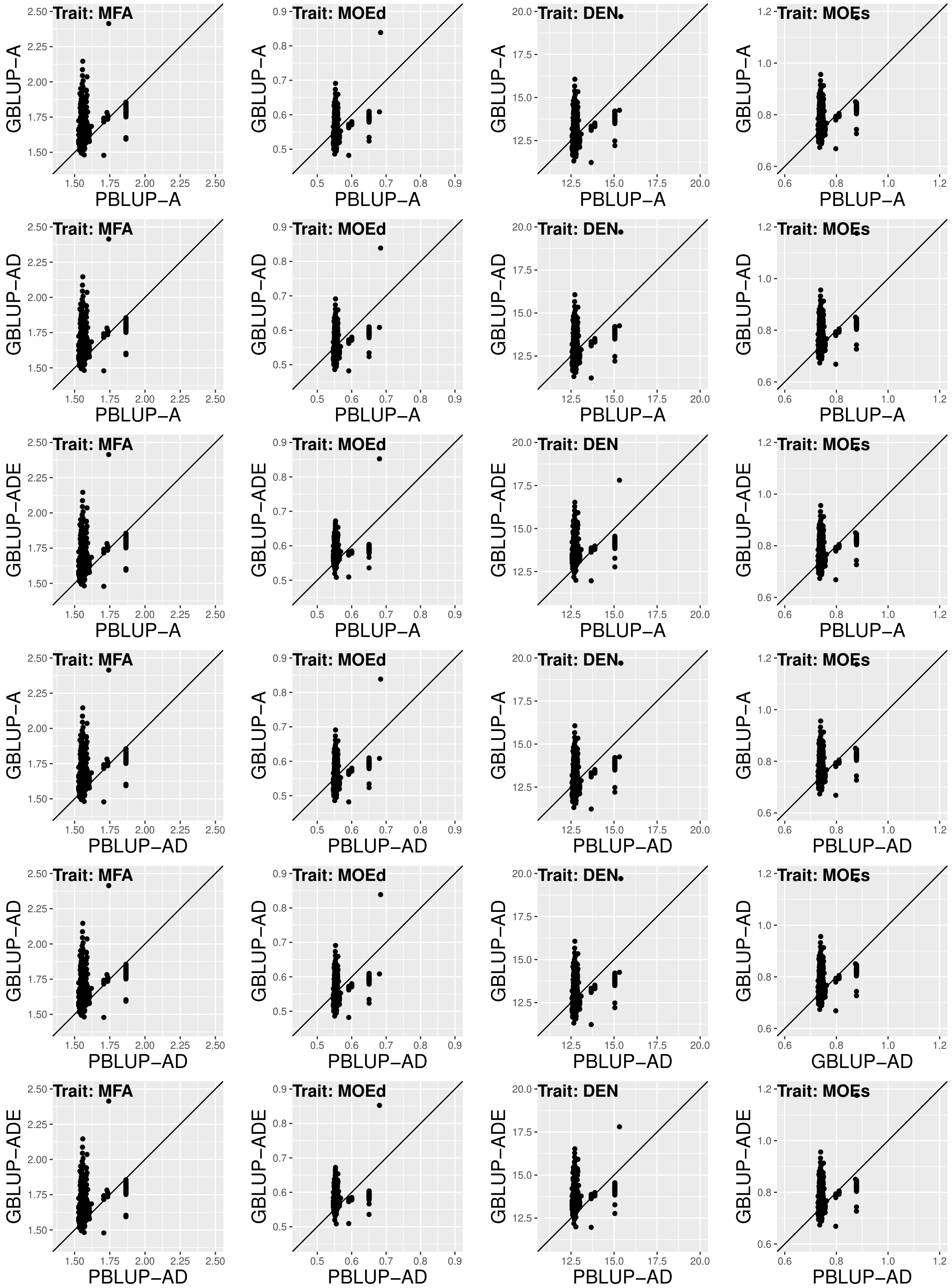

Figure S2. Comparison of Standard Error of Predictions (SEPs) of the estimated breeding- and genetic-values between GBLUP and PBLUP models, for wood traits.

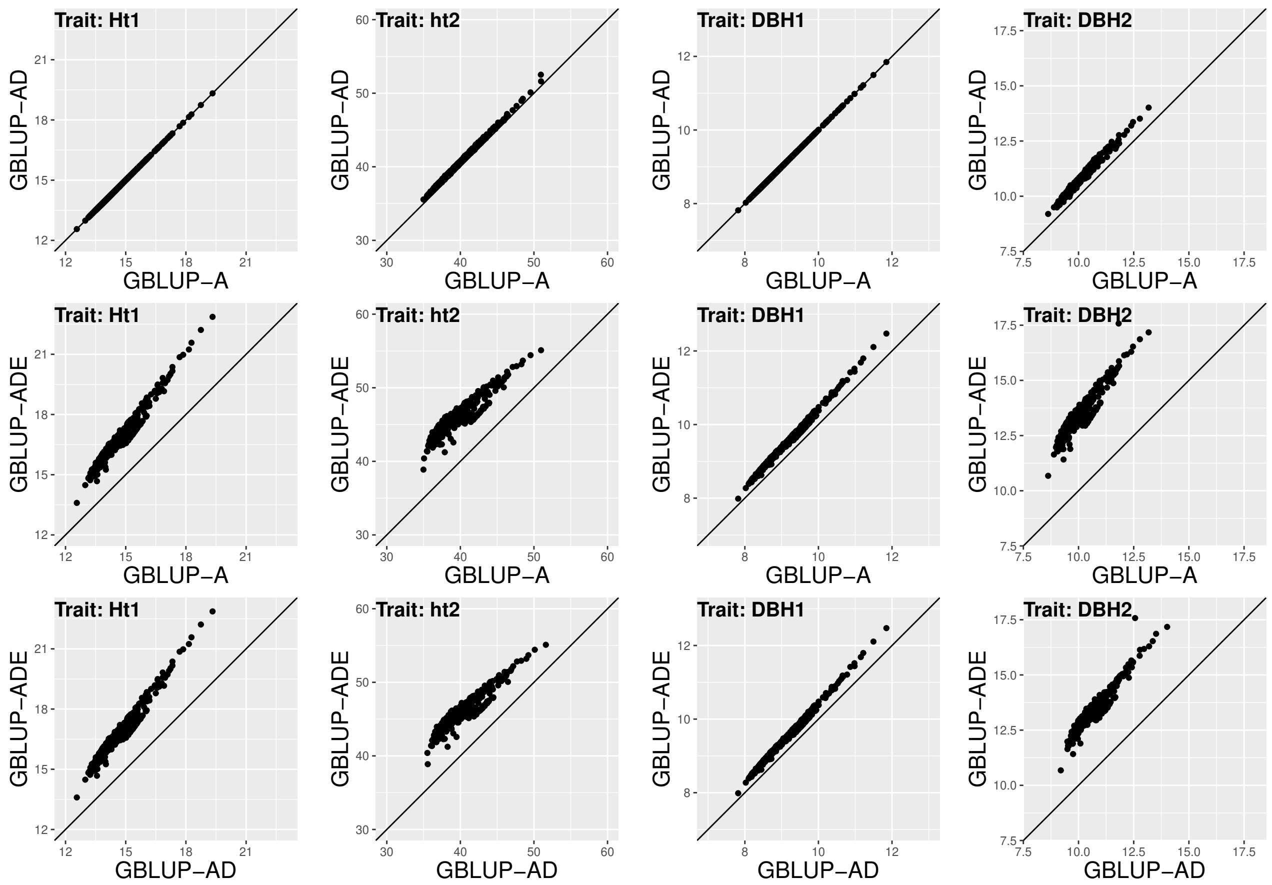

Figure S3. Comparison of Standard Error of Predictions (SEPs) of the estimated breeding- and genetic-values between GBLUP-A, GBLUP-AD and GBLUP-ADE models, for growth traits.

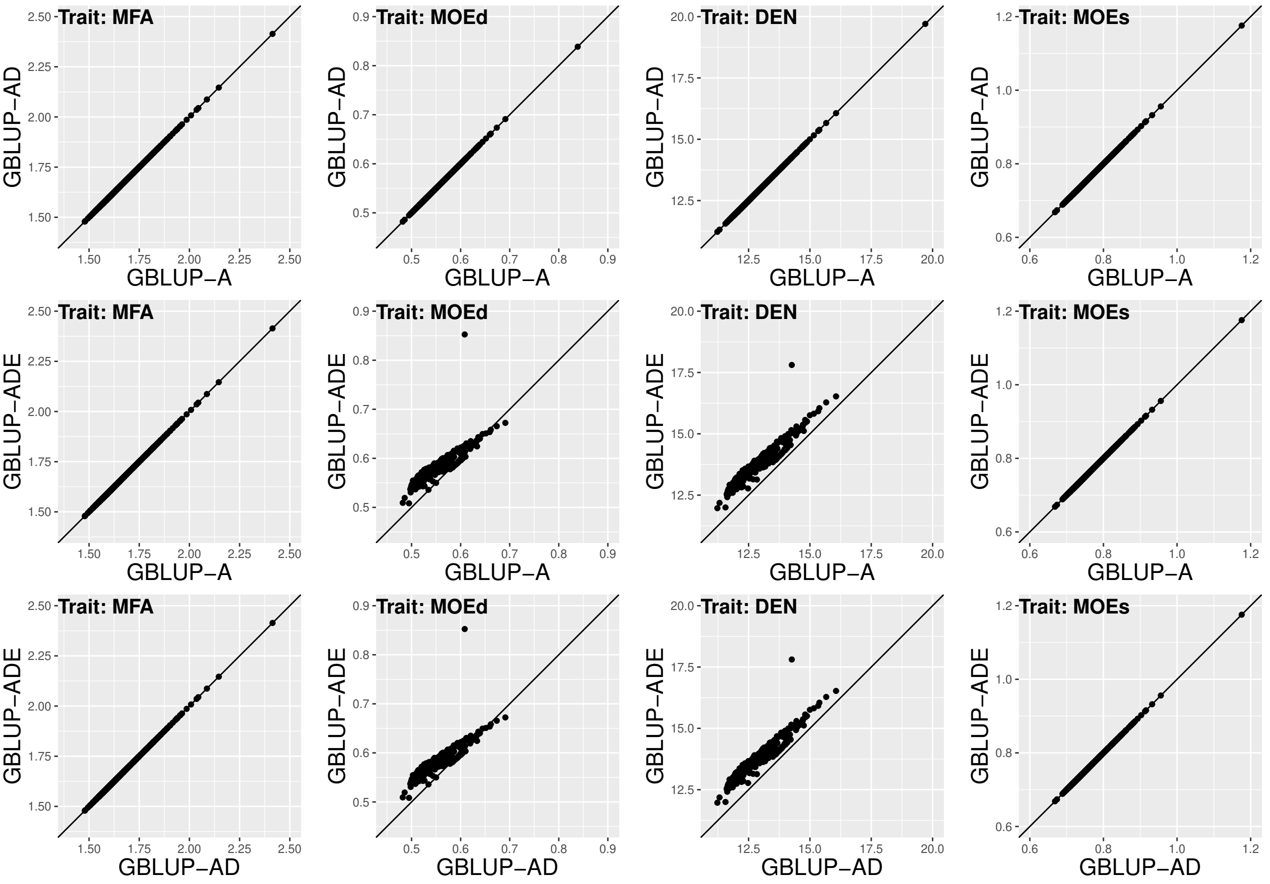

Figure S4. Comparison of Standard Error of Predictions (SEPs) of the estimated breeding- and genetic-values between GBLUP-A, GBLUP-AD and GBLUP-ADE models, for wood traits.

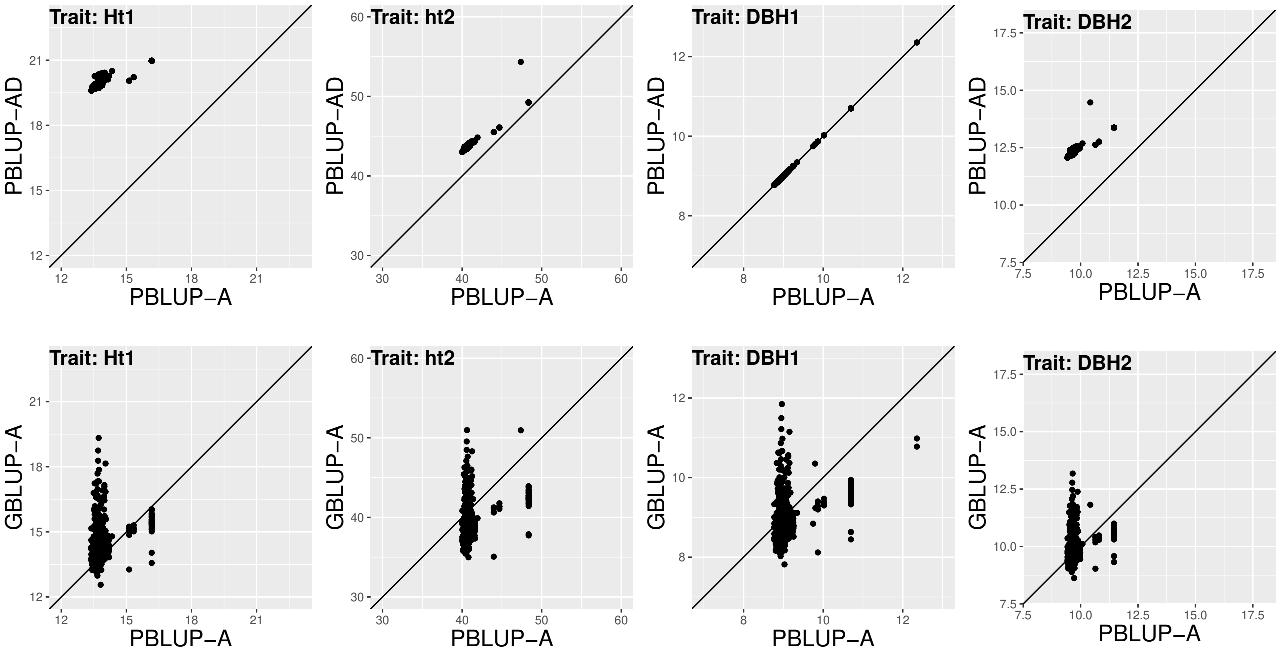

Figure S5. Comparison of Standard Error of Predictions (SEPs) of the estimated breeding- and genetic-values between PBLUP-A and PBLUP-AD models, for growth traits.

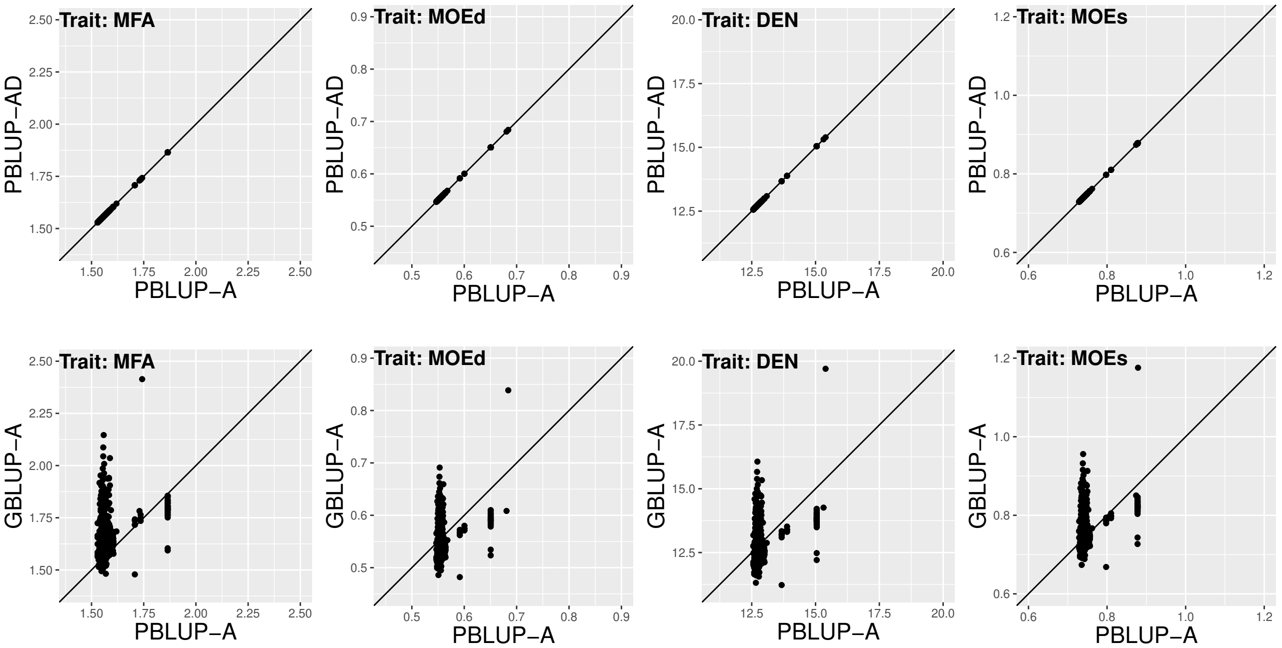

Figure S6. Comparison of Standard Error of Predictions (SEPs) of the estimated breeding- and genetic-values between PBLUP-A and PBLUP-AD models, for wood traits.
